# Research Progress on Non-Destructive Testing Technology and Equipment for Poultry Eggshell Quality

**DOI:** 10.3390/foods14132223

**Published:** 2025-06-24

**Authors:** Qiaohua Wang, Zheng Yang, Chengkang Liu, Rongqian Sun, Shuai Yue

**Affiliations:** 1 College of Engineering, Huazhong Agricultural University, Wuhan 430070, China; yangzhengyzh@webmail.hzau.edu.cn (Z.Y.); ys7lck@163.com (C.L.); srq19860912878@163.com (R.S.); yueshuai@webmail.hzau.edu.cn (S.Y.); 2 Key Laboratory of Agricultural Equipment in Mid-Lower Yangtze River, Ministry of Agriculture and Rural Affairs, Wuhan 430070, China; 3 National Egg Processing Technology Research and Development Branch Center, Wuhan 430070, China

**Keywords:** eggshell quality, eggshell characteristics, non-destructive testing, equipment

## Abstract

Eggshell quality inspection plays a pivotal role in enhancing the commercial value of poultry eggs and ensuring their safety. It effectively enables the screening of high-quality eggs to meet consumer demand for premium egg products. This paper analyzes the surface characteristics, ultrastructure, and mechanical properties of poultry eggshells. It systematically reviews current advances in eggshell quality inspection technologies and compares the suitability and performance of techniques for key indicators, including shell strength, thickness, spots, color, and cracks. Furthermore, the paper discusses challenges in non-destructive testing, including individual egg variations, species differences, hardware precision limitations, and inherent methodological constraints. It summarizes commercially available portable and online non-destructive testing equipment, analyzing core challenges: the cost–accessibility paradox, speed–accuracy trade-off, algorithm interference impacts, and the technology–practice gap. Additionally, the paper explores the potential application of several emerging technologies—such as tactile sensing, X-ray imaging, laser-induced breakdown spectroscopy, and fluorescence spectroscopy—in eggshell quality inspection. Finally, it provides a comprehensive outlook on future research directions, offering constructive guidance for subsequent studies and practical applications in production.

## 1. Introduction

Poultry eggs, as an essential food resource, are widely consumed due to their high nutritional value. To meet the growing demand for poultry eggs, stringent quality control measures must be implemented to ensure that high-quality eggs are delivered to consumers. The eggshell, serving as a natural protective barrier, effectively prevents physical damage and microbial invasion [1]. Therefore, eggshell quality is a critical parameter for assessing overall egg quality. During production and packaging, eggs are subjected to various mechanical impacts and vibrations, possibly leading to shell fractures. Cracked eggshells facilitate the penetration of environmental microorganisms, causing spoilage in table eggs [2]. In the case of hatching eggs, shell defects reduce hatchability and increase embryonic mortality [3]. For eggs used in further processing, compromised shell integrity negatively impacts flavor and texture, thereby reducing their culinary value. Consequently, an intact and clean eggshell is a prerequisite for ensuring the quality and safety of edible eggs, while uniform coloration, smoothness, and the absence of visible defects are also crucial attributes.

Furthermore, eggshell color, strength, and thickness are key indicators of eggshell quality. Uniformly colored eggs are generally preferred by consumers, though regional preferences for eggshell color vary [4]. Higher eggshell strength enhances resistance to mechanical compression and vibration during storage and transportation, thereby reducing the risk of breakage. A thicker and more uniform eggshell provides better protection against microbial contamination, preserving internal egg quality. However, excessively thick shells can hinder the hatching process by making it difficult for chicks to break through, leading to increased embryonic mortality [5]. Therefore, the detection of shell cracks, as well as the prediction of eggshell strength and thickness, plays a crucial role in safeguarding egg integrity, minimizing damage risk, and enhancing both the economic and nutritional value of eggs.

Currently, eggshell quality assessment methods can be categorized into traditional and non-destructive testing (NDT) techniques. Traditional methods involve manual sensory evaluation of eggshell cracks, color, and spots, as well as quasi-static compression tests for strength measurement and micrometer measurement for determining eggshell thickness [6,7,8]. These approaches are labor-intensive, time-consuming, and often destructive, limiting their applicability in large-scale industrial settings. In contrast, non-destructive testing techniques enable the evaluation of eggshell properties without compromising shell integrity or internal contents. These methods encompass acoustic vibration analysis, machine vision, optical inspection, electrical property measurements, ultrasonic testing, and terahertz spectroscopy. Compared to conventional approaches, NDT techniques offer higher efficiency and faster detection speeds. This paper provides a comprehensive review of non-destructive eggshell quality assessment technologies and related equipment, offering researchers a systematic overview of advancements in this field.

## 2. Characteristics of Poultry Eggshells

### 2.1. Surface Characteristics of Poultry Eggshells

As the outer protective layer of an egg, the poultry eggshell serves as a natural mechanical barrier. Its surface characteristics include color, texture, spots, and gloss.

Color plays a crucial role in eggshell quality, food safety, and consumer perception. The color spectrum of poultry eggshells is extensive, with common hues including white, brown, pink, and green. Studies analyzing over 100 types of poultry eggshell colors have identified protoporphyrin, biliverdin, and their zinc chelates as the primary pigments responsible for the diverse eggshell colors [9]. These pigments are deposited on the shell membrane and all layers of the shell, but they are primarily concentrated in the outermost layer of the calcareous shell and the cuticle [10]. Eggshell color varies by poultry species and individual differences, and even within the same species, there can be noticeable variations in eggshell color among different individuals [11]. Research suggests that eggshell color is influenced by genetic factors, diet, and rearing environment [12]. Additionally, studies indicate that genetically determined eggshell color not only affects shell strength, thickness, and hatchability but also influences the overall characteristics of the egg [13].

**Figure 1:**
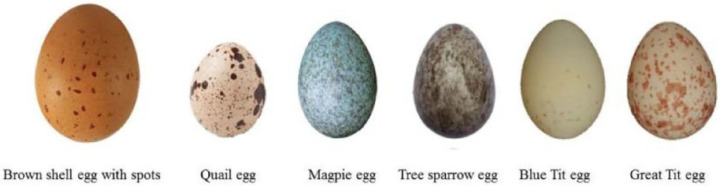
Spotted eggs of different poultry eggs (adapted from ref. [18]).

The surface of poultry eggshells exhibits many stable natural textural features, including spots, stripes, and ridges [14,15]. These features can be classified into physiological and pathological types based on their formation mechanisms. The spots observed on quail and sparrow eggshells are evolutionarily conserved physiological traits, appearing as irregular, dot-like, or blotched pigment deposits, as shown in Figure 1. While the exact formation mechanism of these spots remains unclear, it is hypothesized to be related to mineral deposition and other biological factors during eggshell formation. In the case of chicken eggs, spotted eggs often exhibit abnormal pigmentation, such as brown or white spots, which primarily result from abnormal calcium deposition in the uterus. This abnormality may be caused by oviduct inflammation or imbalanced dietary nutrition, leading to irregular eggshell pigment deposition. Consequently, spotted eggs not only reflect the reproductive health status of poultry but also have significant implications for the egg industry. Gosler et al. [16] first proposed an evaluation system for eggshell spotting using three parameters: pigment intensity, spot distribution, and spot size. Pigment intensity is rated on a scale from 1 (light pigmentation) to 5 (dark pigmentation); spot distribution ranges from 1 (concentrated distribution) to 5 (even distribution); and spot size is scored from 1 (small spots) to 3 (large spots). This evaluation system provides a valuable reference for spot detection in poultry eggs. Furthermore, dark spots, characterized by their irregular, watermark-like appearance, exhibit a translucent nature primarily due to nanoscale structural distortions in the mammillary layer. These spots are widely observed across various types of chicken eggs. Under transmitted light, dark spots typically appear translucent, a phenomenon often referred to as the “translucency effect” of eggshells. The presence of such spots may compromise the permeability and structural uniformity of the eggshell, potentially leading to a reduction in its mechanical strength.

Eggshell glossiness is another critical surface characteristic. Some eggshells appear smooth and glossy, while others exhibit rough or mixed gloss surfaces. The glossiness of an eggshell is influenced by its surface structure, composition, and rearing conditions [17].

### 2.2. Ultrastructure of Poultry Eggshells

**Figure 2:**
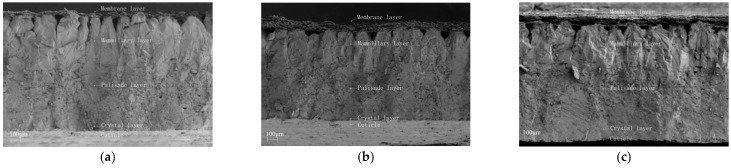
Cross-sections of different eggshells observed by SEM at appropriate magnifications (adapted from ref. [26]). (**a**) Chicken eggshell cross-section (180×). (**b**) Duck eggshell cross-section (150×). (**c**) Goose eggshell cross-section (119×).

The components of eggshells include organic components such as matrix, eggshell membrane, buttons, and cuticle, as well as inorganic components such as calcium carbonate crystals, which account for 94% to 98% of the total weight of the eggshell [19]. The ultrastructure of poultry eggshells is typically analyzed using Scanning Electron Microscopy (SEM) and Transmission Electron Microscopy (TEM). Scanning electron micrographs of cross-sections from various common poultry eggshells (Figure 2) reveal the microstructural characteristics of the eggshell, which consists, from outer to inner layers, of the cuticle layer, crystal layer, spongy layer, papillary layer, and eggshell membrane [20]. The ultrastructure of the eggshell plays a critical role in evaluating eggshell quality, as the inorganic components significantly influence its mechanical properties [21]. Notably, the spongy layer, which constitutes approximately two-thirds of the eggshell’s thickness, is a key determinant of eggshell strength [22,23]. Research has shown that supplementing laying hens’ diets with organic trace minerals containing Mn, Zn, and Cu can enhance the thickness of the spongy layer, thus improving both eggshell thickness and strength [24]. Additionally, the density of the papillary layer is closely correlated with eggshell strength; a higher density results in stronger eggshells [25]. These findings emphasize the significance of eggshell ultrastructure in determining eggshell quality.

### 2.3. Mechanical Properties of Poultry Eggshells

Key indicators for evaluating the mechanical properties of eggshells include shell strength and stiffness. Shell strength, also known as eggshell fracture strength, quantifies the eggshell’s ability to withstand compressive forces under vertical pressure, typically assessed through static compression tests. Stiffness is categorized into static and dynamic types. Static stiffness (K_stat_) measures the degree of bending or deflection the eggshell undergoes when subjected to force, commonly evaluated by placing the egg between two parallel steel plates and applying compression using a universal tensile and compression testing machine, followed by calculating the slope of the force–deformation curve [27]. Dynamic stiffness (K_dyn_), derived from a mathematical mass–spring model, serves as a quantitative indicator of overall shell resistance based on the dynamic behavior of poultry eggs [28]. Coucke et al. [29] utilized the resonance frequency of impact vibration, combined with the egg’s mass and a mathematical model, to compute dynamic stiffness. Their results showed strong correlations between dynamic stiffness, static stiffness, and eggshell thickness, with correlation coefficients of 0.71 and 0.6, respectively. De Ketelaere et al. [27] designed an acoustic testing system to further validate the correlation between dynamic stiffness and other parameters. Their study improved the correlation between dynamic stiffness, static stiffness, and eggshell thickness, while also demonstrating that dynamic stiffness exhibited a correlation coefficient of 0.64 with eggshell fracture strength. These findings highlight the importance of both static and dynamic stiffness as crucial indicators of eggshell mechanical properties. Additionally, eggshell strength and stiffness are closely related to its ultrastructure, particularly the palisade and mammillary layers [30,31]. Therefore, studying the mechanical properties of eggshells is essential for evaluating eggshell quality, ensuring both the integrity and durability of the eggshell.

## 3. The Current Research Status of Poultry Eggshell Quality Detection Technology

### 3.1. Traditional Methods for Eggshell Detection and Evaluation

Common indicators for assessing and detecting poultry eggshell quality include shell strength, thickness, surface spots, color, and crack detection.

(1) Eggshell Strength: Typically evaluated through quasi-static compression tests, which are inherently destructive. The primary instrument used in experimental studies is the eggshell strength tester [32,33].
(2) Eggshell Thickness: Measured using tools such as a micrometer, requiring the eggshell to be broken for accurate assessment.
(3) Eggshell Surface Spots: Traditionally identified through sensory evaluation, where human observers classify spots based on predefined grading criteria.
(4) Eggshell Color: Qualitative analysis involves direct visual observation and comparison with standard color charts, whereas quantitative analysis predominantly employs spectrophotometry. A reflectometer is commonly used to measure eggshell color intensity [34].
(5) Eggshell Crack Detection: Typically performed through visual inspection, with manual grading used to assess crack severity.

In summary, traditional detection methods are highly dependent on manual labor, with drawbacks such as strong subjectivity, low detection efficiency, and destructive testing. These methods fail to meet the requirements for efficient, accurate, and cost-effective detection, and still face technological limitations.

### 3.2. Current Research Status and Comparison of Eggshell Non-Destructive Testing Technologies

Current NDT technologies for assessing poultry eggshell quality primarily include acoustic vibration analysis, computer vision, spectral analysis, electrical signal analysis, and ultrasound. Table 1 provides a detailed comparison of the specific NDT methods employed for different types of detection information. Each technique operates on distinct detection principles: Acoustic vibration analysis evaluates eggshell quality by analyzing acoustic signals collected by sensors. It can be classified into contact and non-contact modes, where relevant signal parameters are extracted and analyzed to identify variations indicative of eggshell quality. Computer vision technology employs cameras as a substitute for human vision to capture images under varying environmental conditions. These images undergo processing for feature extraction and pattern recognition to assess eggshell characteristics. Spectral analysis characterizes eggshell quality by examining differences in light absorption, scattering, or emission properties. Electrical signal analysis indirectly acquires biological information related to eggshell quality by analyzing its electrical properties. Ultrasound technology determines eggshell thickness by evaluating the propagation of ultrasonic waves within the shell. This is achieved by measuring the time interval between the emission of ultrasonic pulses and the reception of reflected waves. Compared with traditional destructive testing methods, these NDT techniques significantly enhance detection efficiency and accuracy while minimizing damage to the eggs.

**Table 1:** Comparison of non-destructive techniques and methods for eggshell quality detection. All images are sourced from the corresponding publications and are licensed under the Creative Commons Attribution 4.0 International (CC BY 4.0) license.

Testing Information	Technical Means	Schematics	Algorithms and Models	Performance	Advantages	Disadvantages
Strength (static stiffness)	Acoustic resonance technology [35]	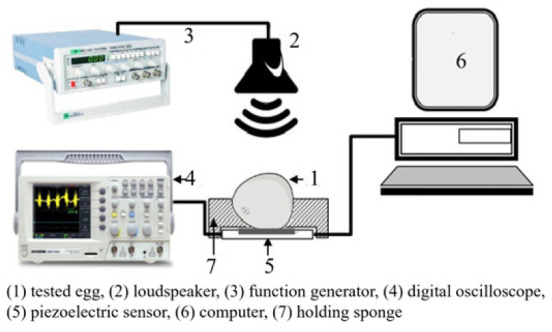	Frequency analysis	The correlation coefficients between the resonance frequency of the eggshell and its strength and thickness are 0.97 and 0.91	Fast and intuitive; strong correlation; capable of simultaneously assessing two information indicators	The resonance effect is highly influenced by environmental factors
	Acoustic impact signals [36]	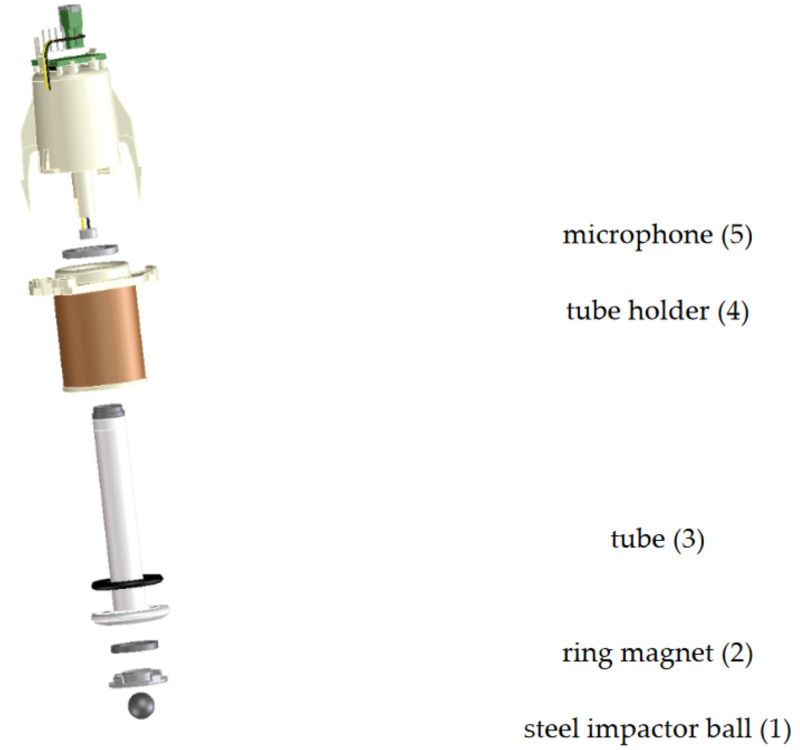	Hertzian contact theory, time-domain signal analysis.	The static stiffness measured by quasi-static compression tests exhibits a correlation of 0.93 with the average stiffness obtained using this method	Simple structure; good correlation	Acoustic signals are highly influenced by the environment
	Hyperspectral imaging [37]	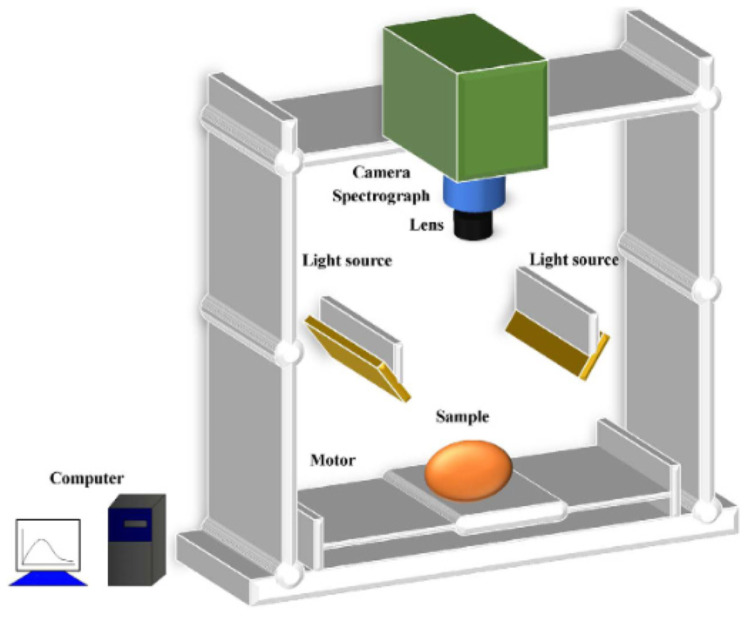	Extraction of characteristic wavelengths for regression coefficients and PLSR modeling	The correlation coefficient between the predicted values and eggshell strength is 0.841	Spectral and image information can enable multi-information detection	The equipment cost is high, and the regression performance is moderate
	Non-destructive load technology [38]	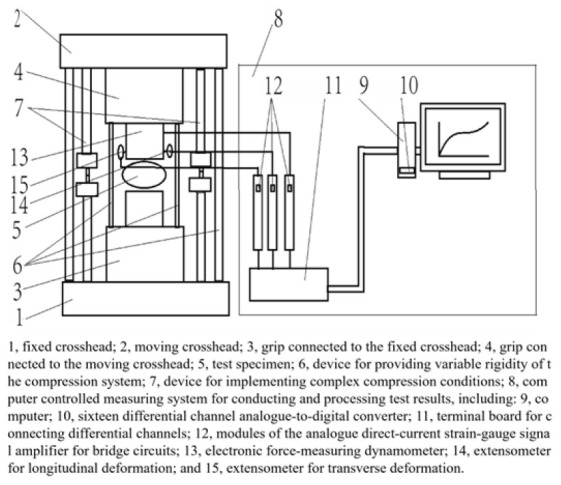	Multifactorial linear equation	Prediction of maximum shell strength (R ≈ 1)	Good prediction effect	The prediction performance relies on the maximum non-destructive load value, and there is potential for damage
Thicknesses	Visible/near-infrared transmission spectroscopy [39]		Preprocessing techniques such as standard normal variate transformation, followed by PLSR modeling for regression analysis	The correlation coefficient for the PLSR prediction set is 0.84, with a standard error of 0.01	The detection is simple and rapid	The shell color has a significant impact; the prediction performance is moderate
	Optical coherence tomography [40]		Quantitative measurement of image data	A measurement resolution with a penetration depth ranging from 7 μm to 1.7 mm was achieved	Convenient, non-destructive, and accurate; capable of obtaining variations in thickness	The equipment cost is high
	Terahertz time-domain reflectance spectroscopy [41]	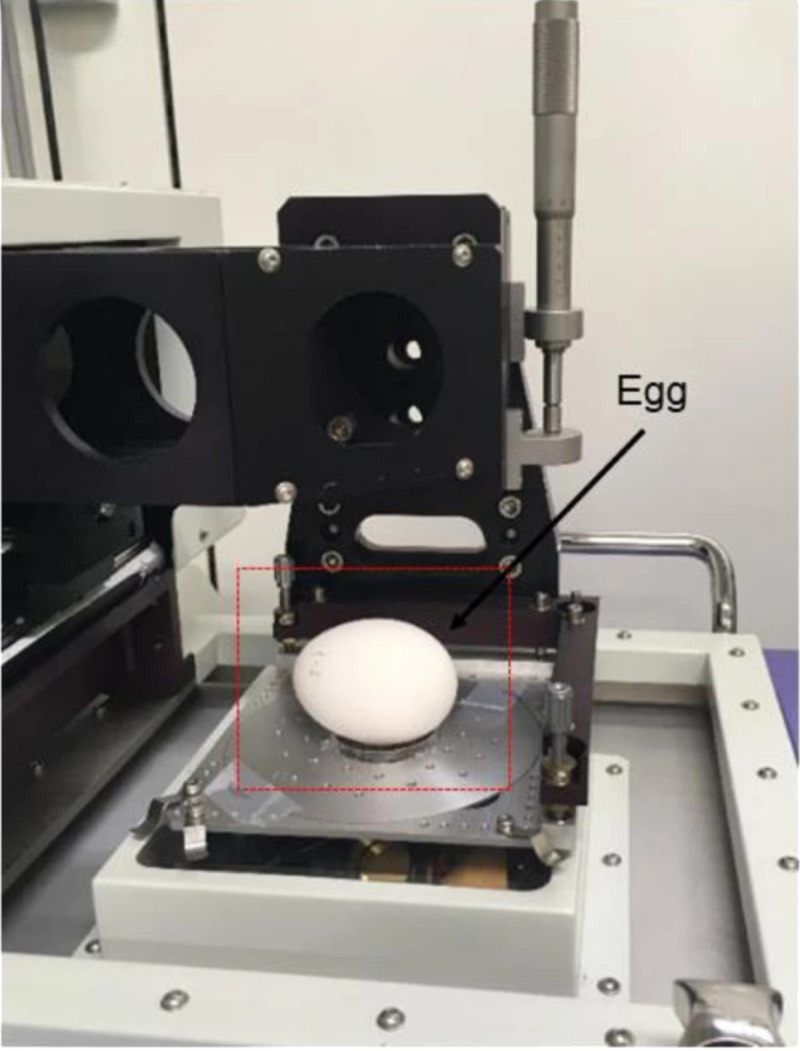	Linear regression	The coefficient of determination (R^2^) of the model is 0.93	Fast and non-destructive; the model performs excellently	The instrument is expensive, and the maintenance costs are high
Color	Visible/near-infrared transmittance spectroscopy [42]	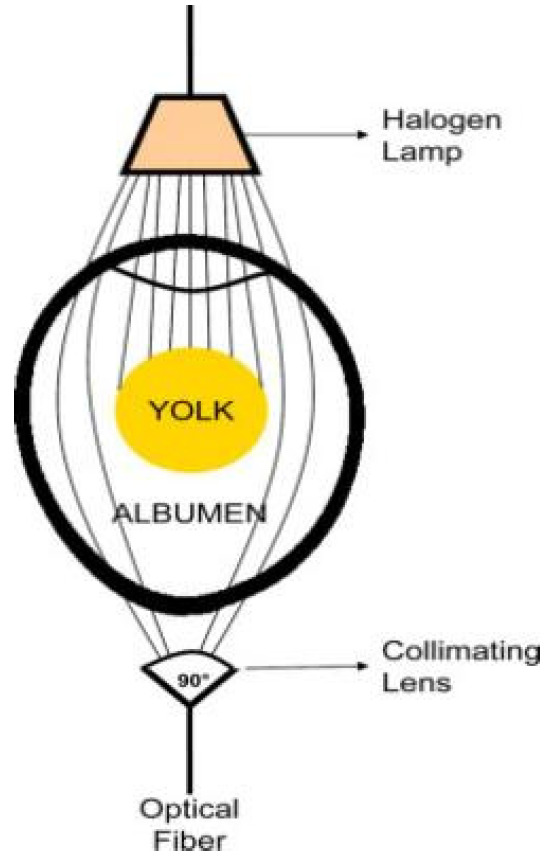	The ratio of relative transmittance at two characteristic spectral wavelengths (TCV)	The TCV value contains more information regarding the actual pigment deposition both inside and on the surface of the eggshell	The obtained pigment information is more comprehensive	It is not possible to fully quantify the external pigment deposition
	Visible/near-infrared reflection spectroscopy [43]	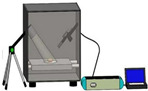	Principal component analysis for band extraction, BP neural network modeling	The values for the test set are Rv = 0.9975, RMSEP = 0.0277, and SEP = 0.0159	Fast and non-destructive; while achieving high classification accuracy, it can also provide information on eggshell strength	The implementation of online detection incurs high costs
Speckles	Machine vision [14]	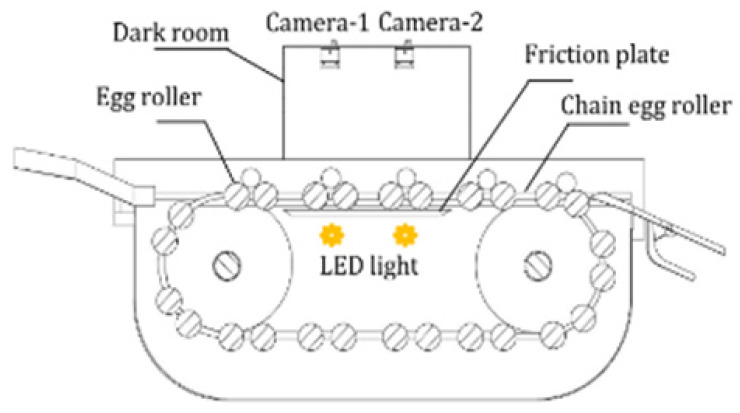	Image processing algorithms	The processing speed of the speckle image is 1 frame per 0.5 s	Quickly and accurately calculate the distribution of dark spots and the ratio of the projected area of dark spots	The fit between the calculated number of dark spots and the manual count is slightly poor
Crackles	Acoustic vibration [44]	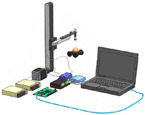	Cross-correlation analysis and Bayesian classification method	The crack detection accuracy reaches 97%, with a false rejection rate of 1%	The operation is straightforward, and the classification performance is satisfactory	It is susceptible to environmental noise interference
	Machine vision [45]	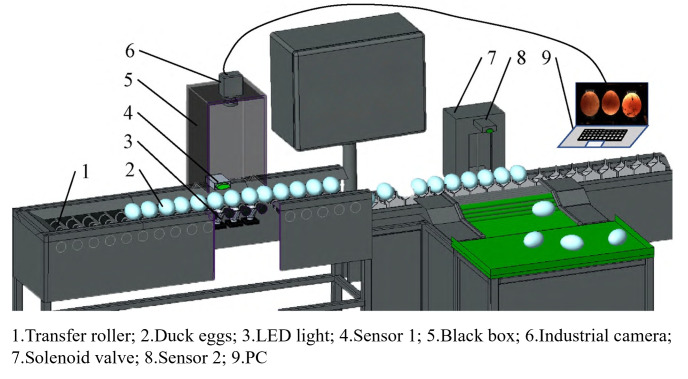	Improved EfficientNetV2 model	The crack recognition accuracy is 98.03%, with a detection time of 6.61 ms	Achieving batch processing of rapid detection for dirty and cracked eggs on the production line, with high classification performance	Sensitive to light source
	Visible/near-infrared reflection spectroscopy [43]	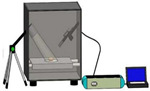	Principal component analysis for band extraction, followed by modeling using a backpropagation neural network	The crack classification accuracy for brown-shell, green-shell, and white-shell eggs are 100%, 100%, and 98.75%	Fast, non-destructive; high classification accuracy	The influence of eggshell color needs to be considered
	Fourier transform near-infrared [46]		selection of VIP feature wavelengths and PLSR modeling for regression	The RMSE, RPD, and R^2^ of the validation set are 0.82 N, 5.62, and 0.90	Fast, green, and non-destructive	The color of the eggshell and the glossiness of its surface have a significant impact
	Hyperspectral imaging [47]	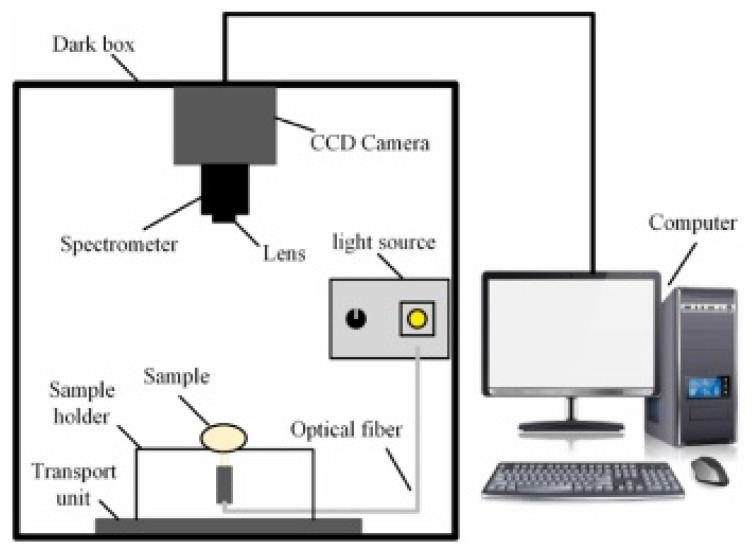	XGBoost classification model	The crack detection accuracy is 93.33%	While analyzing other information from spectral data, image information was also utilized for crack detection	The instrument cost is high; relying solely on imaging data makes it unsuitable for crack detection
	Bioelectrical signals [48]	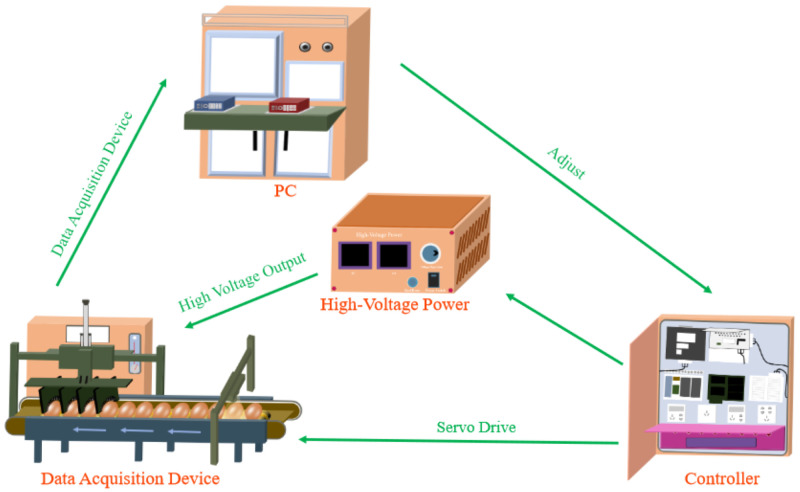	Wavelet scattering transform for feature extraction and convolutional neural network modeling for classification	The crack detection accuracy exceeds 99%	Fast and real-time; high detection accuracy	Highly influenced by voltage; requires precise control of the voltage range

#### 3.2.1. Eggshell Strength and Thickness Detection

Eggshell strength and thickness detection hold significant research and application value in the field of poultry science and engineering. The main research technologies currently include acoustic vibration, ultrasound, spectral techniques, hyperspectral imaging, optical coherence tomography (OCT), and non-destructive compression techniques.

(1) Acoustic vibration technology

Acoustic vibration technology is extensively employed in the industrial online inspection of eggshell quality due to its rapid response characteristics and straightforward signal processing capabilities. It is composed of three primary components: the excitation module, the signal acquisition module, and the signal processing module [49]. The signal acquisition module can be divided into contact and non-contact methods, with their respective structures, advantages, and disadvantages outlined in Table 2. The contact method typically employs acceleration sensors or piezoelectric sensors, which are closely coupled with the egg to detect changes in the object’s acceleration and convert these variations into electrical signals. Lin et al. [50] and Sun et al. [51] applied partial least squares (PLS) models to analyze the frequency response of eggshells under mechanical excitation and developed eggshell strength prediction models through various frequency domain feature extraction algorithms, achieving satisfactory results. In comparison, the non-contact method meets the needs for fast, non-destructive testing, particularly in online detection applications. Non-contact methods generally use miniature microphones to capture the acoustic vibration signals generated by mechanical excitation. These signals are then amplified, filtered, and processed to detect eggshell quality. El Attar et al. [35] studied the relationship between the resonance values of eggshells and their strength and thickness. The experimentally measured resonance values showed correlation coefficients of 0.97 and 0.91 with eggshell strength and thickness, respectively, highlighting the applicability of the acoustic excitation method as a non-contact testing approach to evaluate egg strength in poultry production lines.

In recent research, De Ketelaere et al. [36,52] achieved high prediction accuracy in assessing eggshell static stiffness by integrating Hertzian contact theory with analysis of impact contact duration and velocity characteristics. The combined approach of Hertz contact theory and acoustic signal acquisition offers a fast and cost-effective solution for non-destructive eggshell static stiffness evaluation, thereby offering an alternative reliable characterization technique for eggshell strength.

**Table 2:** Different types of signal acquisition methods in acoustic vibration technology. All images are sourced from the corresponding publications and are licensed under the Creative Commons Attribution 4.0 International (CC BY 4.0) license.

Signal Acquisition Methods	Sensor	Acquisition Devices	Advantages	Disadvantages
Contact type [53]	Acceleration sensor, piezoelectric sensor	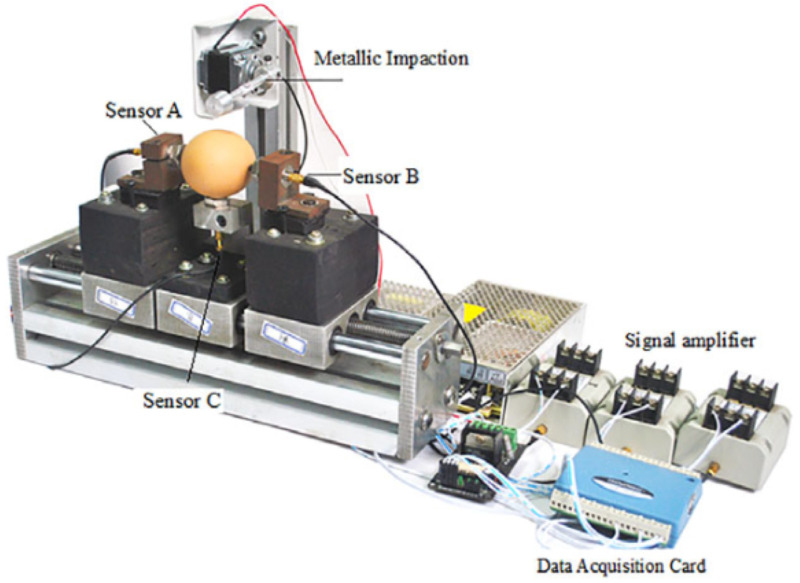	High detection sensitivity, wide frequency range	The weight of the sensor affects the vibration of the eggs, and can easily cause damage to the eggs
Non-contact type [54]	Microphone	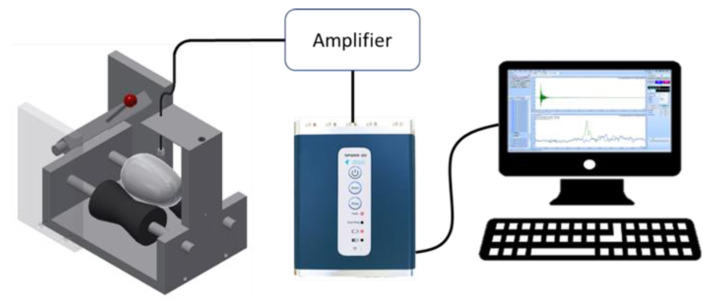	Simple structure and cost-effective	Highly susceptible to environmental noise

(2) Ultrasonic technology

Ultrasound consists of mechanical sound waves that originate from the oscillatory motion of molecules within a propagation medium. Due to its high frequency, reaching up to 20 kHz, ultrasound is imperceptible to the human ear [55]. Consequently, ultrasonic technology has been increasingly applied in non-destructive testing within the food industry. Early studies have demonstrated that ultrasound can be effectively used for the non-destructive measurement of eggshell thickness. The principle involves analyzing ultrasonic signals transmitted into the eggshell and calculating the time delay between the emitted ultrasonic pulse and the received reflected wave to determine the eggshell thickness [56]. This principle has been commercialized, and currently, ultrasonic thickness gauges available on the market are also utilized in scientific research. A prominent example is the Eggshell Thickness Gauge (ESTG-01, Orka Technology Ltd., Ramat HaSharon, Israel) [57,58,59,60]. Comparative studies between ultrasonic thickness measurement and traditional thickness measurement methods have revealed a significant correlation between the two, with the highest heritability observed for thickness measurements taken at a 45° angle from the large end of the egg [61,62,63]. While this technique faces dual constraints in practical application—operational reliance on contact-based measurement and coupling agents compromising detection efficiency, alongside economic limitations from elevated equipment costs restricting large-scale implementation—it maintains high precision and robust repeatability in thickness measurement. Consequently, it retains significant practical utility in experimental research and targeted implementation contexts.

(3) Spectral analysis technology

As a rapid and non-destructive detection method, spectral analysis has been widely applied in eggshell quality assessment in recent years, demonstrating certain advantages in predicting eggshell strength and thickness. Xiong et al. [64] explored the application of near-infrared (NIR) spectroscopy in eggshell quality assessment and developed a predictive model for eggshell quality based on near-infrared diffuse reflectance spectra. The results indicated that the partial least squares regression (PLSR) model, established within five characteristic wavelength ranges and preprocessed using multiple scattering correction, achieved the best predictive performance for eggshell strength, with a correlation coefficient of 0.86. The PLSR model for eggshell thickness yielded a correlation coefficient of 0.81. Additionally, visible/near-infrared (VIS/NIR) spectroscopy is another commonly used spectral analysis method. Dong et al. [39] employed VIS/NIR transmission spectroscopy to measure eggshell thickness and processed the data within an effective wavelength range of 480–960 nm. The PLS model achieved correlation coefficients of 0.86 and 0.84 for the calibration and prediction sets, respectively, with standard errors of 0.01. Similarly, Ahmed et al. [46] utilized Fourier transform near-infrared (FT-NIR) spectroscopy combined with PLSR to predict eggshell strength. In the validation set, the PLSR model using 10 selected wavelengths achieved a root mean square error (RMSE) of 0.82 N and a coefficient of determination (R^2^) of 0.90. In contrast, terahertz (THz) technology—a relatively novel spectroscopic analysis method—demonstrates particular suitability for detecting crystalline structures, yet remains underutilized in non-destructive eggshell quality assessment. Khaliduzzaman et al. [41] employed THz reflection spectroscopy to estimate eggshell thickness by analyzing the vibrational distance in the frequency domain and establishing a predictive model. The results indicated a coefficient of determination (R^2^) of 0.93, a root mean square error of prediction (RMSEP) of 0.009, and a resolution of less than 10 μm.

Spectral technology has demonstrated significant potential in eggshell quality assessment. Various spectral methods, including NIR spectroscopy, VIS/NIR spectroscopy, and FT-NIR spectroscopy, have been effectively combined with PLSR models to achieve reliable predictions of eggshell strength and thickness. Concurrently, THz wave technology emerges as an advanced spectroscopic approach, demonstrating exceptional predictive capability in thickness assessment owing to its high resolution and sensitivity to crystalline materials. Although currently constrained by substantial instrumentation costs and predominantly confined to fundamental research, this technique shows potential to evolve into a robust tool for avian eggshell characterization.

(4) Optical imaging technology

Optical imaging technology utilizes the principles of light reflection, refraction, and other optical properties to capture structural and functional information of objects. In recent years, some researchers have attempted to apply hyperspectral imaging (HSI) technology to eggshell quality assessment. Xie et al. [37] employed hyperspectral imaging to measure eggshell strength, achieving correlation coefficients of 0.835 and 0.841 using PLS and RC-PLS models, respectively. Additionally, Sabuncu et al. [40] utilized OCT to obtain high-resolution OCT scan images at 930 nm, which enabled accurate calculation of eggshell thickness with high precision. By capturing structural and functional details with high accuracy, optical imaging technology offers a reliable solution for eggshell quality assessment. Whether in the prediction of eggshell strength and crack detection using hyperspectral imaging or in the precise measurement of eggshell thickness using optical coherence tomography, optical imaging has demonstrated significant potential in eggshell quality research.

(5) Non-destructive compression technology

Non-destructive compression technology involves the application of static compression to eggs without causing breakage, enabling the collection of compression curves and parameters for analyzing their correlation with eggshell quality. As early as 1962, researchers developed an instrument based on this method to assess eggshell strength non-destructively, establishing a correlation between eggshell strength and its non-destructive deformation values, ranging from 0.59 to 0.88. Building upon this research, Voisey et al. [65,66,67] conducted numerous experiments, improving analytical tools, increasing non-destructive testing loads, and optimizing shell compression speed. Their data confirmed that the relationship between eggshell deformation under selected non-destructive forces and fracture force during quasi-static compression is nonlinear. Narushin et al. [38] employed extremely low compression speeds and five non-destructive load points to measure eggshell deformation, successfully calculating eggshell strength. Their findings suggest that this method can be used to develop effective computational programs for non-destructive eggshell strength evaluation. The utilization of deformation parameters for non-destructive prediction of eggshell strength remains a key research focus in poultry science and engineering.

#### 3.2.2. Eggshell Crack Detection

Eggshell crack detection has long been a focal point in the industry, with various detection methods offering distinct advantages and limitations. Significant advancements have been achieved in terms of detection accuracy and sensitivity across multiple techniques, including acoustic vibration, computer vision, spectral analysis, and electrical signal analysis.

(1) Acoustic vibration technology

Acoustic vibration technology demonstrates high practical applicability in eggshell crack detection, particularly in identifying micro-cracks imperceptible through visual inspection. By mechanically exciting specimens and analyzing vibrational responses, this technique precisely captures subtle structural anomalies. Current research focuses on excitation methodologies, yielding four principal approaches: tapping vibration, magnetostrictive frequency-sweeping, inclined-rolling vibration, and electromagnetic excitation—each exhibiting distinct advantages in detection performance, system complexity, and apparatus design (see Table 3).

Tapping vibration represents the most prevalent implementation due to its structural simplicity and integration feasibility. Multi-sensor systems measuring shell vibration signals effectively detect cracks while reducing impact frequency and cost [68]. Wang et al. [69] achieved 96% crack detection accuracy using miniature microphones to capture post-impact acoustic signatures. Xu et al. [70], Yumurtaci et al. [71], and Balci et al. [72] similarly analyzed acousto-mechanical signals, with the latter two attaining near-perfect (≈100%) crack identification. Acoustic resonance theory further enhanced percussion-based detection: Cheng et al. [73] and Sun et al. [74] reported 98% and 95.5% accuracy, respectively. Lai et al. [54] and Kertész et al. [75] improved micro-crack sensitivity through frequency-domain analysis and feature extraction. System configuration significantly influences performance; Lashgari et al. [76] optimized response stability using 45° plastic-ball excitation with 180° microphone placement, while Sun et al. [44] determined the ideal actuator mass for efficacy–safety balance. Magnetostrictive frequency-sweeping employs concentrated, controllable forced vibration to enhance the signal-to-noise ratio (SNR). Systems developed by Ding et al. [77], Zhang et al. [78], and Luo et al. [79] achieved >95% discrimination between intact and cracked eggs. Lu et al. [80] subsequently refined signal processing, attaining 98% accuracy for intact specimens. Inclined-plane rolling utilizes gravitational self-excitation for low-cost, simplified systems. Jin et al. [81] reported 90% crack recognition using acoustic signatures from eggs rolling down seven-step inclines. Comparative analysis by Lashgari et al. [82] indicated that percussion impulse response (IR) outperforms inclined-plane (IP) signals in accuracy, though IP remains economically advantageous. Electromagnetic excitation generates repeatable stimuli via current-driven magnetic fields, enabling >95% crack detection accuracy when integrated with acoustic acquisition [83]. Current limitations include unilateral shell excitation, necessitating design optimization.

Collectively, despite variations in precision, complexity, and apparatus design, all excitation methods demonstrate high diagnostic accuracy (typically >90%). This confirms the significant potential of acoustic vibration technology for non-destructive crack detection, while highlighting optimization requirements for industrial-scale implementation.

**Table 3:** Different types of excitation modules in acoustic vibration technology for eggshell quality detection. All images are sourced from the corresponding publications and are licensed under the Creative Commons Attribution 4.0 International (CC BY 4.0) license.

Incentive Methods	Incentive Devices	Advantages	Disadvantages
Tapping vibration method [72]	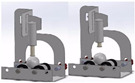	Short excitation time, simple structure, and low cost	The excitation repeatability is poor, requiring control over the striking force
Magnetostrictive frequency sweeping vibration method [80]	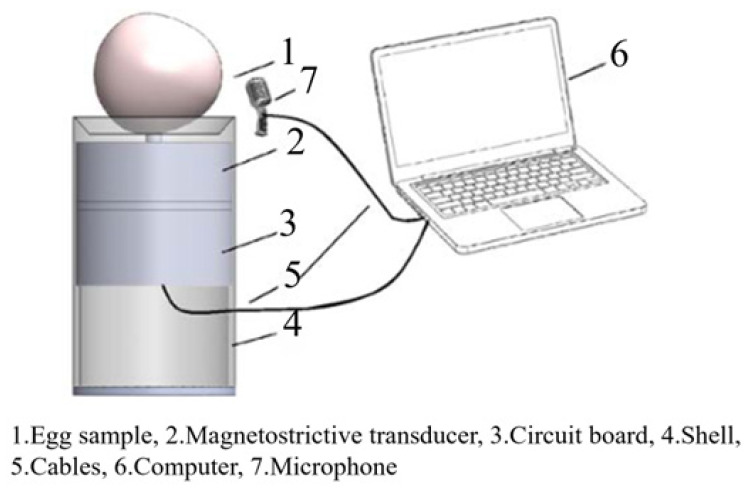	Good excitation repeatability and a high signal-to-noise ratio	The excitation process is time-consuming
Inclined plate rolling vibration method [81]	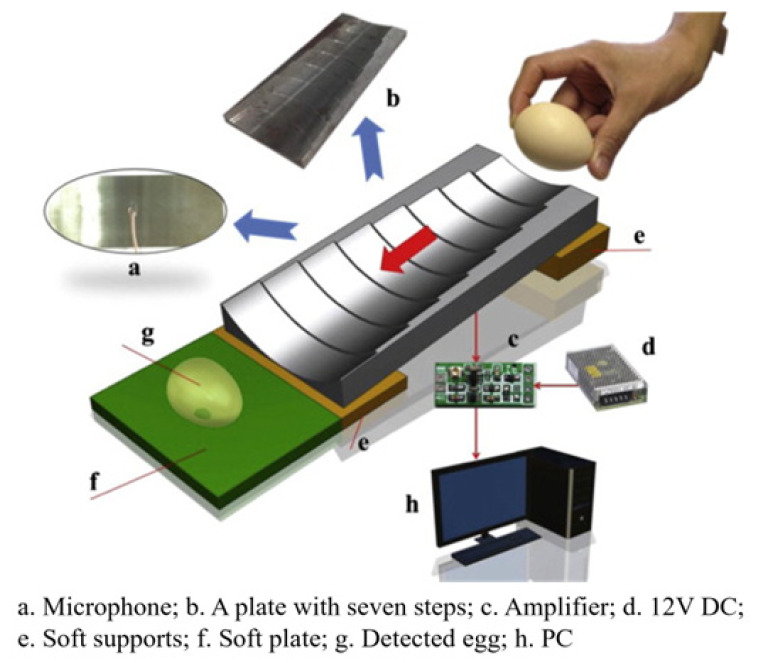	The structure is simple and cost-effective	The damage rate is relatively high, and the time consumption is prolonged
Electromagnetic excitation method [83]	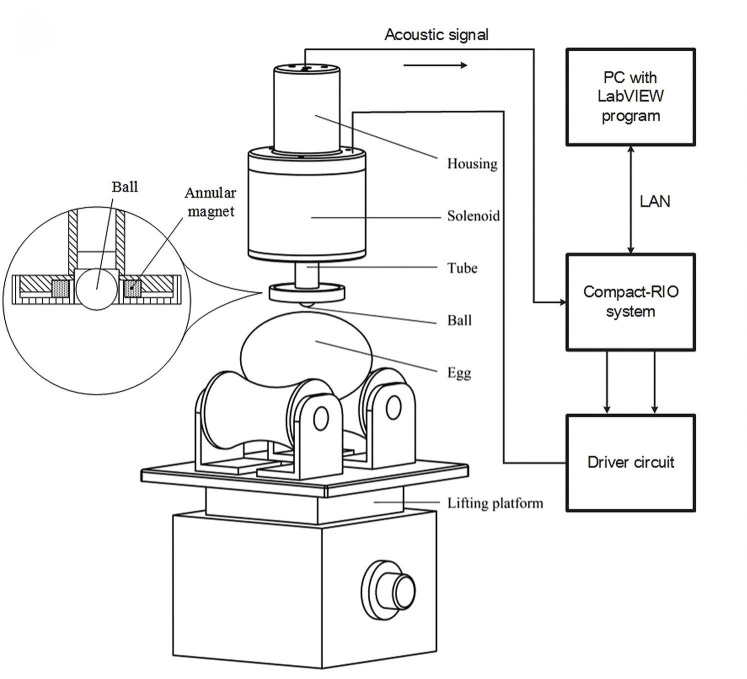	The system features a fast response time, high accuracy, and integrates both the sound collection module and the excitation module into a single unit	Prolonged on/off cycles can lead to heat generation, and the circuit and control system are relatively complex

(2) Computer vision technology

Computer vision technology is primarily employed for detecting surface cracks in eggshells. Leveraging high-resolution imaging and image processing algorithms, it identifies micro-cracks imperceptible to the naked eye. Although limited in detecting internal subsurface defects, this approach demonstrates high accuracy and technical maturity in surface inspection applications.

To enhance crack saliency, researchers extensively adopt image enhancement and feature extraction algorithms. Sun et al. [84], Guan et al. [85], and Chen et al. [86] have employed sequential wave signal extraction, negative LOG operator, and laser enhancement techniques, respectively, to process image data and improve crack detection accuracy. To further enhance detection precision, Sun et al. [87] optimized sequential wave signal extraction and recognition algorithms, Zhang Jian et al. [88] applied the particle swarm optimization algorithm, and Tu Weihù et al. [89] refined the Canny operator using an improved locust optimization algorithm, all of which significantly improved detection performance. In addition to accuracy, detection efficiency is critical. Priyadumkol et al. [90] improved an automated machine vision system for crack detection in continuously rotating eggs, facilitating the rapid collection of full-surface images. Abbaspour-Gilandeh et al. [91] achieved a detection time of 0.7 s per egg, while Sun et al. [92] introduced an innovative adaptive lighting system for real-time crack detection.

Classification models employing traditional machine learning methodologies—such as Support Vector Machines (SVMs) and artificial neural networks (ANNs)—are extensively employed for crack identification based on visual features. Abdullah et al. [93], Wu et al. [94], and Hao et al. [95] all employed SVM models to distinguish intact eggs from cracked ones, achieving a maximum detection accuracy of 98.75%. Mota-Grajales et al. [96] utilized structured light scanning combined with an artificial neural network (ANN), reaching a crack detection accuracy of 97.5%.

In recent years, deep learning has further advanced the performance of crack detection. Several researchers have adopted convolutional neural networks (CNNs) for eggshell crack detection, yielding better classification results than SVMs, with an accuracy of 95.38% [97]. Wong et al. [98] proposed a pre-trained CNN model based on the AlexNet architecture for eggshell feature extraction, demonstrating performance comparable to human evaluators and highlighting CNN’s potential in handling highly non-uniform speckle patterns. Selecting the most suitable CNN model is crucial. Li Shu et al. [99] tested YOLO-v5, ResNet, and ShuffleNet for crack detection, achieving validation accuracies of 98.8%, 97.8%, and 99.4%, respectively. Botta et al. [100] used a fine-tuned DenseNet121 model, achieving a detection accuracy of 98.38%. To meet the demands of industrial applications, Tang et al. [101], Wang et al. [45], and Tang et al. [102] developed real-time crack detection methods, ensuring both high accuracy and rapid detection speeds. Turkoglu et al. [103] introduced a deep learning-based real-time machine vision system, integrating a pre-trained residual network with a bidirectional long short-term memory (BiLSTM) model, achieving an accuracy of 99.17%.

(3) Spectral analysis technology

Spectral analysis is a non-contact detection method that identifies cracks by analyzing the spectral response of eggshells across different wavelengths. Han et al. [43] applied VIS/NIR reflectance spectroscopy, combined with PCA-BP modeling, to assess egg integrity, extracting characteristic wavelength bands at 1100–1260 nm, 1648–1698 nm, and 2380–2410 nm. The classification accuracy for pink, green, and white eggshells reached 100%, 100%, and 98.75%, respectively. Spectroscopic techniques demonstrate irreplaceability in high-end scenarios requiring simultaneous accommodation of eggshell colorimetric variations and micro-crack detection. However, their technical complexity and substantial cost impose significant constraints on practical implementation for crack identification applications.

(4) Optical imaging technology

Hyperspectral imaging technology, by integrating spatial and spectral information (typically encompassing 256–512 continuous bands), demonstrates a unique advantage in crack detection through multidimensional parameter joint analysis. Xie et al. [37] employed hyperspectral imaging to analyze eggshell strength while simultaneously detecting cracks, achieving classification accuracies of 97.06% and 88.24% with PLS-DA and RC-PLS-DA models, respectively. Yao et al. [47] utilized hyperspectral imaging in conjunction with an extreme gradient boosting (XGBoost) classification model to identify cracked eggs, attaining an accuracy of 93.33%. Hyperspectral imaging is typically used as a supplementary indicator, enhancing both detection accuracy and efficiency when combined with other analytical parameters.

(5) Electrical signal analysis technology

In recent years, Electrical signal analysis technology based on dielectric response theory exhibits distinct advantages in the detection of submicron-scale cracks. Shi et al. [104] designed an eggshell micro-crack detection system that extracts time-domain, frequency-domain, and wavelet-domain features from current signals, classifying cracked eggs with an accuracy exceeding 99% using machine learning algorithms. Subsequently, Shi et al. [48] further refined their detection system, improving micro-crack detection accuracy while reducing system voltage and controlling current levels to prevent egg damage. Their application of a wavelet scattering convolutional network further enhanced detection precision. Additionally, Joe et al. [105] developed an innovative crack detection device utilizing discharge phenomena. This system, comprising a customized high-voltage power supply, flexible electrode needles, and a rotating mechanism, enables the comprehensive 360-degree examination of each egg, overcoming the limitations of existing detection technologies. These studies provide a novel non-destructive testing approach for eggshell quality assessment, demonstrating significant applicability and broad potential in practical implementations.

#### 3.2.3. Eggshell Color and Spot Detection

The formation mechanisms of eggshell color and speckles are closely associated with pigment deposition. Detection technologies based on similar principles have evolved into two major approaches: spectroscopic analysis and computer vision. The former relies on precise optical measurements to achieve quantitative analysis of subtle color variations, while the latter employs intelligent image processing to meet the requirements of industrial-scale grading.

(1) Computer vision technology

Computer vision technology constructs a multidimensional detection system by integrating high-resolution imaging modules, intelligent image processing algorithms, and deep learning models. This system enables the simultaneous extraction of eggshell color features and dynamic analysis of speckles. Puente et al. [106] captured images of eggs from different angles and used image processing software to compute the speckle area ratio to estimate speckle content. Gomez et al. [107] developed a software tool named SpotEgg, which automatically captures and analyzes eggshell color and speckles, including their quantity, size, distribution, and shape, establishing a standard for computer vision-based speckle detection. Wang et al. [14] proposed an automated quantitative detection method based on machine vision, enabling the rapid evaluation of the number and area proportion of dark speckles on eggshells. Jiang et al. [108] applied a GoogLeNet-based approach to classify eggs with and without dark speckles, achieving a detection accuracy of 98.19%.

(2) Spectral analysis technology

Spectral analysis detects subtle differences in eggshell color and speckles by analyzing the reflected and transmitted light spectra interacting with the eggshell material. Spectrophotometry, one of the earliest methods for measuring eggshell color and speckles, remains widely used. By analyzing the characteristics of reflected spectra, spectrophotometers calculate eggshell color values, typically represented by tristimulus values to describe brightness, red-green hue, and yellow-blue hue [109,110]. Holveck et al. [111,112] used reflectance spectroscopy to calculate the spectral chromaticity and brightness of speckles. Han et al. [43] employed VIS/NIR reflectance spectroscopy combined with a BP neural network to develop an eggshell color sorting model. In addition to reflectance spectroscopy, transmittance spectroscopy has also been utilized for eggshell color measurement. Mertens et al. [42] introduced a novel method based on VIS/NIR transmittance spectroscopy, analyzing eggshell color using the transmission color ratio (the transmittance at 643 nm divided by the transmittance at 610 nm).

Through systematic review and comparative analysis, various eggshell quality detection technologies exhibit significant differentiated characteristics and applicable scenarios: Acoustic vibration technology, through multiple excitation methods and high detection convenience, effectively captures abnormalities in the microstructure of eggshells and reveals their physical properties, making it particularly valuable for detecting shell cracks and physical characteristics at a relatively controlled cost. Crucially, calibrated excitation parameters constitute a fundamental prerequisite for system stability and high-accuracy detection. These parameters require precise calibration according to eggshell biomechanical properties to prevent inadequate signal acquisition or specimen compromise. Computer vision technology, leveraging high-resolution imaging, deep learning models, and image processing algorithms, accurately detects cracks, color, and speckle features, making it well-suited for large-scale automated inspection on production lines. Spectral technology analyzes eggshell optical properties across different wavelengths, providing a non-destructive solution for detecting cracks, strength, and thickness. However, it involves a relatively complex operational process, requiring an extended preheating period for data acquisition, which limits its suitability for continuous conveyor-based inspection. Hyperspectral imaging technology integrates spectral and imaging data advantages, offering comprehensive characterization for multi-parameter assessments. Ultrasonic technology, benefiting from high-precision measurement and mature signal acquisition, is particularly advantageous in cases where destructive testing is not feasible. Terahertz wave technology, as an emerging detection method, features non-contact and high-sensitivity properties, making it a promising avenue for eggshell thickness measurement, though its high equipment cost remains a challenge. Non-destructive compression testing, with improvements in load parameters and compression speed, continues to enhance its precision and applicability; however, its adaptability to high-throughput detection is limited, requiring precise control over compression speed and loading points, making experimental procedures complex. Electrical signal analysis, as an innovative non-destructive detection paradigm, introduces a novel perspective for eggshell quality assessment. For instance, newly developed devices based on discharge phenomena enable 360-degree comprehensive inspection, overcoming the limitations of traditional methods and laying the foundation for further research on eggshell detection based on electrical signal principles. Nevertheless, the implementation of such technology necessitates sophisticated hardware design, including high-voltage power sources and flexible electrode needles, presenting a higher technical threshold.

### 3.3. Conformity Assessment of NDT Methodologies with International Eggshell Quality Standards

Eggshell quality constitutes a critical metric for the commercial grading and safety regulation of eggs. Regulatory bodies globally prioritize “structural integrity” and “surface cleanliness” as fundamental criteria for premium-grade classification. Chinese national standards stipulate that AA, A, and B grade eggs must have clean, intact shells with natural coloration and no visible contaminants. In the United States, the grading system mandates that AA and A grades must have clean, unbroken, and normal shells, while permitting minor stains on B grade shells provided structural integrity remains intact. Similarly, the European Union specifies that A grade eggs must have normally shaped, clean, and undamaged shells, with non-conforming specimens classified as B grade. Evidently, “crack-free integrity” and “cleanliness” represent universal prerequisites in global egg grading frameworks. NDT methodologies provide essential technical support for egg grading through their efficiency, precision, and non-invasive nature. Acoustic vibration technology detects incipient defects—including subsurface micro-cracks—by capturing subtle structural responses to external excitation, thereby directly addressing integrity compliance requirements. Computer vision and optical imaging technologies excel in the automated identification of surface contaminants and fine fractures, corresponding precisely to cleanliness and integrity standards. Exploratory multimodal integration of acoustic and visual/optical techniques presents significant potential: acoustic signals facilitate internal anomaly detection while visual data characterizes surface conditions, collectively enhancing crack detection comprehensiveness and operational robustness. This synergy proves particularly valuable for complex specimens and confidence-level augmentation.

In summary, mainstream NDT methods demonstrate strong compatibility with major international standards in terms of crack detection and surface condition assessment. Future research may focus on enhancing system integration capabilities, standardizing detection sensitivity, and improving regulatory adaptability across regions. Such efforts would support the broader application of NDT technologies in global egg quality monitoring and regulatory frameworks.

## 4. Challenges in the Development of Non-Destructive Testing Equipment for Poultry Eggshell Quality

### 4.1. Issues in Eggshell Quality Detection

Currently, significant progress has been made in the non-destructive detection of poultry eggshell quality, including crack detection, strength and thickness measurements, and color and spot identification. However, several challenges and limitations still persist.

(1) Individual variability of eggs: Different poultry eggs exhibit variations in shape, shell color, and surface texture, which require the detection technology to be adaptable to these individual differences. Technologies with poor adaptability tend to result in lower detection accuracy.
(2) Hardware precision limitations: While visible cracks on the eggshell are relatively easy to identify, detecting small cracks and invisible cracks with complex shapes, which are not discernible to the naked eye, demands higher hardware precision. Therefore, balancing detection accuracy and cost is critical.
(3) Limitations of detection methods: Existing non-destructive testing methods, such as spectral analysis, still struggle to match the precision of destructive tests for measuring eggshell thickness and strength. The performance of different detection techniques varies, making it crucial to select the most appropriate method based on the specific requirements of the task.
(4) Species-specific influences: Unlike other agricultural products such as apples, pears, or kiwis, which experience changes in hardness over time and can be measured at multiple points using destructive testing, the strength of poultry eggs only undergoes a single measurement due to its one-time nature. This makes it challenging to identify inherent natural patterns in the strength of eggs.

### 4.2. Research Status and Analysis of Detection Equipment

In recent years, significant advancements have been made in the non-destructive testing technology for poultry eggshell quality. This not only reflects the growing demand in the poultry egg market but also highlights the increasing emphasis on the development of non-destructive testing technologies for egg quality. To meet consumer demand for high-quality eggs, the variety and functionality of related detection equipment have been continuously innovated and improved.

Eggshell thickness measurement: The eggshell thickness gauge developed by ORKA Technology Ltd., Israel, has undergone multiple technological upgrades, employing ultrasonic measurement technology and high-precision sensors to rapidly measure eggshell thickness without causing any damage. The sensitivity of the device reaches up to 0.001 mm. Additionally, the thickness gauge provides real-time measurement data, which can be transmitted to a computer or cloud platform for further analysis via specialized software. Similarly, the eggshell thickness gauge developed by RobotmationCo., Ltd., based in Tokyo, Japan, utilizes dual-wave ultrasonic technology and features user-friendly operation. The user simply needs to place the probe on the eggshell surface, and the measurement result is immediately displayed on the screen with an accuracy of 0.01 mm.

Eggshell strength measurement: The Dutch company MOBA has developed a groundbreaking eggshell strength detection system. This detection module relies on electromagnetic excitation to capture sound signals, enabling the determination of the eggshell strength quality grade for each egg. It can differentiate and separate weak-shelled eggs from strong-shelled eggs, and can be integrated as a detection module in poultry egg sorting industrial equipment. The Belgian company INDUCT has designed the high-throughput egg quality measurement device. This system captures acoustic signals generated by multiple impacts using a microphone, and analyzes and develops a vibration response equation to calculate the dynamic stiffness of the eggshell.

Eggshell crack detection: The Dutch company MOBA has developed an artificial vision-based eggshell crack detection system, which integrates artificial intelligence and visual technology. By training on a large dataset of eggshell crack images, it enables the detection of hairline cracks on eggs with various shell colors. This detection system, serving as a universal detection module, can be integrated into various MOBA poultry egg sorting machines to conduct systematic quality assessments of poultry eggs. The Japanese company Nabel has developed a crack detection device, which can strike each egg from multiple directions continuously. The device utilizes Fourier transform to process the sound signals generated by the impact on the eggshell, achieving an accuracy rate of up to 95% for cracked egg detection. This system is suitable for high-speed processing environments, with a maximum throughput of 120,000 eggs per hour.

Eggshell color and spot detection:

(1) Portable equipment. The colorimeter developed by Konica Minolta (Japan) employs reflective spectral analysis to output standard colorimetric values such as CIELAB, enabling objective quantification of eggshell color. With a measurement area of 8 mm, the device calculates color values based on the characteristics of the reflected spectrum, expressing them as tristimulus values (XYZ) or CIELAB values (Lab*). When paired with a data processor, it allows for data display, transmission, and printing. In China, the Beijing Tianxiang Feiyu Technology Co., Ltd. has designed a digital speckle evaluation instrument that utilizes transmitted imaging from multiple orientations (large end, small end, left side, and right side) of the egg to assess the overall speckle level and achieve digital recording.
(2) Automated sorting equipment. The high-end egg grading machines developed by SANOVO Technology Group (Denmark) are equipped with integrated color recognition modules capable of effectively distinguishing between brown and white eggs. The system automatically directs eggs to corresponding packaging lanes and separates those with undesired shell colors. Furthermore, it can group brown eggs with uniform tonal characteristics, thereby enhancing grading efficiency and consistency.

In conclusion, with the continuous development of non-destructive testing technologies in the poultry egg industry, the types of detection equipment on the market have gradually diversified, and their functionalities have been continuously optimized, allowing them to better meet various detection needs. In the fields of eggshell thickness, cracks, color, and spot detection, various technological devices have matured. Whether it is portable instruments or automated sorting systems, each demonstrates its unique advantages.

### 4.3. Summary of the Current Situation and Challenge Analysis

The current non-destructive testing technologies for poultry eggshell quality exhibit a dual-track development characterized by “industrial efficiency” and “laboratory precision”. Technology paths centered around computer vision and acoustic vibration, with their comprehensive advantages in detection speed, hardware cost, and scene adaptability, have become the mainstream choice for industrial applications. In contrast, spectral technologies, particularly hyperspectral methods, perform excellently in material composition analysis and deep feature detection but remain confined to laboratory research due to limitations in data dimensionality, equipment cost, and sensitivity to environmental factors. This technological divergence reveals four core contradictions in existing devices:

(1) Paradox between cost and accessibility: Contemporary non-destructive testing systems predominantly remain cost-prohibitive. Hyperspectral implementations incur substantial procurement and maintenance expenses due to core components including full-spectrum illumination sources, cryogenically cooled detectors, and precision control modules. Simplified configurations employing near-infrared (NIR) or multispectral technologies, while structurally streamlined, still necessitate custom-engineered illumination systems with stable performance, uniform irradiance, and extended longevity—yielding considerable lifecycle costs. Even acoustic and vision-based systems require premium data acquisition and processing hardware to achieve high frame rates, micron-scale spatial resolution, or microsecond-scale temporal response, thereby escalating total capital expenditure.
(2) Contradiction between speed and precision: Vision and acoustic technologies achieve industrial-level detection efficiency through hardware innovations (e.g., 1280 fps high-speed cameras, microsecond-level delay acoustic sensors). However, due to the limitations of single-modal perception, there are bottlenecks in analyzing deep indicators such as eggshell thickness distribution and hidden cracks. Although spectral technologies can overcome detection dimensionality through multi-band fusion (e.g., full spectral coverage from 400 to 2500 nm), the complexity of the equipment (requiring integration of spectrometers, temperature control modules, and motion platforms) leads to significantly higher energy consumption and operational costs per test, surpassing the tolerable threshold for industrial scenarios.
(3) Limitations of interference and algorithms: Visual systems are prone to false detection when exposed to fluctuations in lighting or changes in the egg’s position. Acoustic devices, under production line vibrations and background noise, experience a degradation in the effective signal-to-noise ratio. Hyperspectral technologies, with hundreds of feature dimensions, rely on deep neural networks for feature dimensionality reduction, but the time required for model training and the hardware computational demands limit real-time processing. Traditional machine learning algorithms, although computationally efficient, lack sufficient capability for fitting nonlinear relationships.
(4) Disjunction between technology and application scenarios: Current visual and acoustic equipment has significant shortcomings in flexible sorting, miniaturization deployment, and tolerance to extreme environmental conditions. The market urgently requires portable devices that can perform multi-parameter detection (such as simultaneous analysis of cracks, thickness, and strength) with a low operational threshold. However, existing technologies are limited by sensor integration and battery life capabilities.

## 5. Prospects for the Future of Non-Destructive Testing of Poultry Eggshell Quality

The current non-destructive testing technologies for various indicators of eggshell quality have, to a significant extent, mitigated the limitations associated with manual inspection, thereby enhancing detection speed and evaluation efficiency. However, there is still considerable room for improvement in terms of detection accuracy and processing speed, as well as the degrees of automation and intelligence. Further technological advancements are imperative. Therefore, it is crucial to conduct an in-depth exploration of the feasibility and potential applications of emerging technologies and to systematically forecast future development directions.

### 5.1. Emerging Applications of Tactile Sensing Technology

Tactile sensing technology relies on tactile sensors to acquire information when the sensor makes contact with an object, such as contact status, force, and mechanical properties. It has been widely applied in agricultural robots for grasping agricultural products, controlling the applied force to prevent damage to the products. Tactile sensors are mainly divided into two types: rigid and flexible. Early rigid tactile sensors typically consisted of multiple piezoelectric sensor arrays, capable of detecting two-dimensional contact force distribution and hardness distribution, thereby assessing the contact force, hardness, and surface texture of objects [113,114,115]. With technological advancements, flexible sensors, which are made from soft materials, possess characteristics such as flexibility, elasticity, transparency, and multifunctionality. These sensors can capture more tactile information while reducing the potential damage to the measured object [116,117,118,119].

In the field of agricultural product hardness detection, flexible sensors have become essential tools. Bandyopadhyaya et al. [120] designed a flexible tactile sensor that, when integrated with a robotic arm, captures continuous tactile information during the grasping process to classify the softness and hardness of objects, providing new directions for agricultural product hardness detection. Zhang et al. [121] proposed a method for recognizing fruit and vegetable hardness based on tactile array information, using real-time tactile sequence data generated by a robotic hand to train a model. The accuracy of online recognition using PCA-SVM reached 90%. Erukainure et al. [122,123] developed their tactile sensors and, based on instantaneous tactile sensor data and machine learning, established a hardness prediction model for kiwifruit, offering new insights for rapid hardness detection. These cases can serve as valuable references for the quality inspection of poultry eggshells.

With the development of deep learning, the application of optical tactile sensors in hardness estimation has gradually demonstrated advantages. These sensors capture continuous tactile images of the interaction between flexible sensors and objects through visual modules and use deep learning to construct nonlinear models relating tactile images to object hardness. The GelSight sensor, a commonly used optical stereo vision tactile sensor, can detect the compression and deformation shape of the object being measured and has been applied in tomato hardness detection [124]. Lin et al. [125] designed a tactile image acquisition system and, using a CNN-LSTM joint learning network model, detected kiwifruit hardness, achieving more accurate detection. If flexible tactile sensing technology is applied to eggshell quality assessment, it is expected to lead to breakthrough advancements in this field.

### 5.2. Breakthrough in AI-Driven X-Ray Imaging Technology

**Figure 3:**
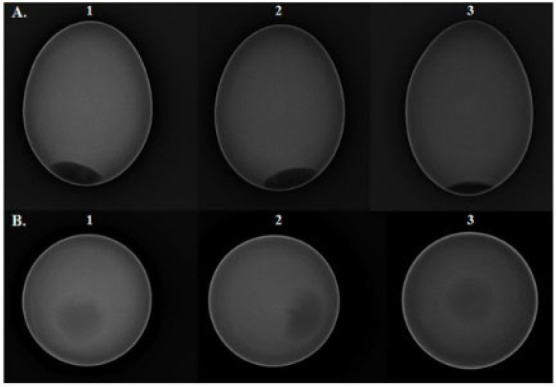
Different quality egg X-ray imaging (reproduced from ref. [128]). (**A**) median and (**B**) axial plane images. (1) special organic eggs, (2) general organic eggs, and (3) conventional eggs.

X-ray imaging technology utilizes X-rays to penetrate objects and generate images on a detector. Due to its strong penetrative capability, it has been widely applied in medical diagnostics and industrial non-destructive testing [126]. In the field of eggshell quality assessment, researchers have employed X-ray imaging to obtain high-resolution images of internal structures. Ray et al. [127] and Hsiao et al. [128] reconstructed two-dimensional and three-dimensional images of chicken and ostrich eggshells, respectively, using X-ray micro-computed tomography (CT), thereby capturing detailed structural information. Studies have shown that in X-ray digital imaging, a distinct boundary exists between the eggshell and its internal contents, as illustrated in Figure 3.

However, achieving high-resolution imaging with X-ray technology relies on advanced CT systems, leading to significant equipment acquisition and maintenance costs. Thus, developing novel image reconstruction algorithms to enhance X-ray imaging quality while reducing system costs has become a critical research focus. With the rapid advancements in artificial intelligence (AI) and large-scale models, AI-driven image reconstruction has demonstrated substantial potential in preserving image fidelity while minimizing radiation exposure and optimizing noise texture. These advancements have already shown remarkable success in CT, MRI, and various X-ray imaging applications across both medical and industrial domains [129]. In the future, integrating AI techniques into X-ray image reconstruction is expected to enhance the resolution and accuracy of eggshell quality assessment while simultaneously lowering equipment costs, providing a more efficient and cost-effective solution for non-destructive eggshell quality evaluation.

### 5.3. Exploration of Laser-Induced Breakdown Spectroscopy and Fluorescence Spectroscopy Technologies

**Figure 4:**
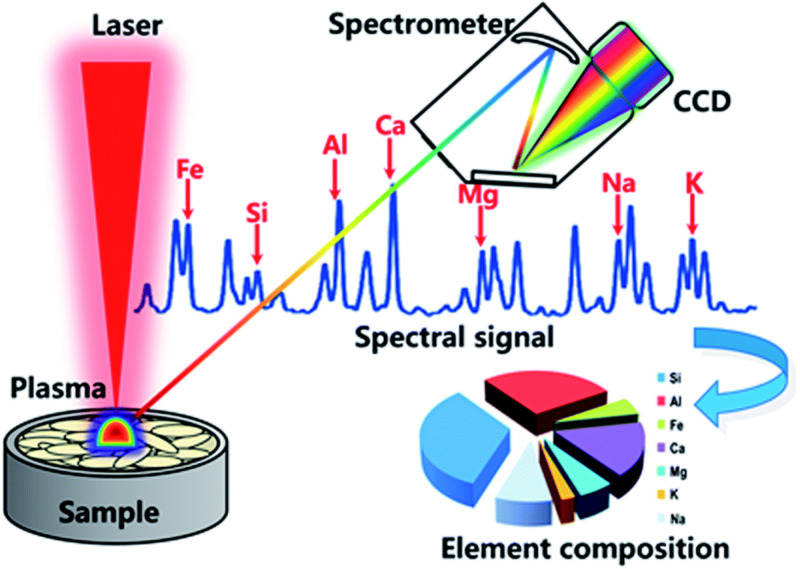
Schematic diagram of laser-induced breakdown spectroscopy technology (reproduced from ref. [133]).

As an innovative branch of atomic emission spectroscopy, laser-induced breakdown spectroscopy (LIBS) has broken through the limitations of traditional detection methods on sample forms and can directly analyze solid, liquid, and gas samples [130]. Its technical principle is shown in Figure 4. It induces high-temperature plasma on the sample surface through high-energy laser pulses and uses a spectrometer to collect the atomic emission spectra radiated by the plasma, thereby achieving simultaneous detection of multiple elements. With its characteristics of minimal damage, rapidity, and simultaneous detection of multiple elements, LIBS technology has demonstrated significant advantages in the detection of minerals, nutrients, and surface contaminants in agricultural products and has become one of the cutting-edge technologies in food safety and material analysis.

In recent years, LIBS technology has been widely applied in the research of agricultural product quality detection. For instance, Ali et al. [131] utilized LIBS technology to track the changes in elemental composition during the ripening process of tomatoes. By analyzing the content patterns of trace and major elements at different ripening stages, they successfully predicted the hardness changes in tomatoes. Additionally, some studies have qualitatively explored the composition of eggshells (including C, O, H, Ca, Mg, Fe, Al, K, Si, and S) using LIBS technology [132]. These studies indicate that LIBS technology shows significant feasibility and innovative potential in the detection of eggshell components and quality assessment. From micro-component analysis to macro-mechanical property prediction, this technology can be used to explore the relationship between key components of eggshells and their mechanical strength, and it can also effectively detect harmful elements remaining on the eggshell surface to ensure food safety. Therefore, LIBS technology can provide a new research approach for non-destructive detection of eggshell quality.

In addition, fluorescence spectroscopy, as an optical technique operating at the molecular level, provides a valuable complement for the non-destructive detection of organic components and microstructural features in eggshells. By detecting fluorescence responses emitted by specific organic constituents—such as porphyrin pigments and aromatic amino acids—under light excitation, this technique demonstrates high sensitivity and molecular specificity, offering promising prospects for the non-invasive quality assessment of poultry eggs. Studies have shown that pigments within the eggshell, particularly protoporphyrin IX, exhibit pronounced red fluorescence when present in monomeric form, while fluorescence intensity markedly decreases upon pigment aggregation or structural disorder, reflecting changes in eggshell composition and organization. Additionally, proteinaceous components in the shell, including tryptophan and tyrosine, emit blue fluorescence under ultraviolet excitation, enabling visualization of the distribution and integrity of the organic matrix [134]. Compared with conventional reflectance or transmittance spectroscopy, fluorescence spectroscopy offers superior detection sensitivity and richer structural information. With the continuous advancement of fluorescence imaging techniques and intelligent analytical algorithms, this method holds great potential for rapid, non-contact detection of microcracks, structural looseness, and pigment distribution anomalies, and is expected to become a key technology for improving the accuracy and efficiency of eggshell quality assessment in poultry production.

### 5.4. Comprehensive Development Directions and Technological Outlook

As consumers’ concerns about food safety and quality continue to grow, the requirements for poultry egg quality have become increasingly stringent. Therefore, refined eggshell quality inspection has become a key factor in selecting high-quality egg products and enhancing market value. This demand is driving the poultry egg industry toward factory-based and intelligent development, creating an urgent need for intelligent eggshell quality monitoring systems that integrate advanced technologies to improve inspection precision and speed, achieve comprehensive quality control, and enhance production efficiency.

(1) Improving technological precision and efficiency: Existing detection equipment may face issues with data accuracy during high-speed operation, especially in micro-crack inspection systems, where it is challenging to ensure high precision while obtaining perfect sound and visual data. Future developments should introduce high-precision, lightweight deep learning algorithms and efficient data analysis methods, combined with hardware optimization, to further accelerate detection speed and enhance data processing capabilities.
(2) Enhancing adaptability in complex environments: Poultry egg production environments may include complex conditions such as noise, dust, and light changes. Visual and optical technologies are susceptible to the influence of ambient light, while acoustic technologies are prone to interference from environmental noise, which can reduce the stability and accuracy of detection technologies and equipment. In the future, integrating visual, acoustic, and optical technologies to form multifunctional, integrated detection systems will improve equipment’s adaptability to complex environments.
(3) Reducing system cost to promote technological adoption: The high overall cost of efficient NDT systems has hindered their widespread adoption and commercialization. Future efforts should focus on developing cost-effective spectral solutions based on low-cost light sources (e.g., LEDs), integrating low-power AI processing modules, and streamlining hardware design. These strategies are expected to significantly reduce system costs and facilitate broader industrial implementation.
(4) Developing portable non-destructive testing instruments: Enterprises typically need to conduct regular sampling inspections of poultry eggs during production to ensure quality control, while research laboratories often require small-batch, diversified sample testing to improve research efficiency. In the future, there will be a need to develop more compact, portable, and easy-to-operate non-destructive testing instruments to meet broader market demands. For example, handheld or miniaturized portable testing instruments can quickly evaluate key indicators such as eggshell thickness and strength.
(5) Establishing multi-model frameworks and integrated databases: Eggshell quality in poultry is influenced by a variety of factors, including breed, rearing environment, and geographical origin. These multidimensional variations introduce significant challenges in model training and generalization across visual, acoustic, and spectral detection modalities. To enhance the robustness and applicability of NDT systems, it is imperative to construct tailored multi-model frameworks and establish integrated databases that encompass diverse breeds, environmental conditions, and production regions.
(6) Building comprehensive traceability systems: As consumer demand for transparency regarding product origins and quality increases, future developments will need to seamlessly integrate eggshell quality information into comprehensive traceability systems. This can be achieved through Internet of Things technologies to record real-time detection data for each egg, linking this information to traceability platforms. This will enable full-quality monitoring of poultry eggs from production to sale, further optimizing supply chain management and enhancing food safety assurance.

## Data Availability

No new data were created or analyzed in this study.
